# The impact of the 2019/2020 Australian landscape fires on infant feeding and contaminants in breast milk in women with asthma

**DOI:** 10.1186/s13006-023-00550-8

**Published:** 2023-02-23

**Authors:** Tesfalidet Beyene, Graeme R. Zosky, Peter G. Gibson, Vanessa M. McDonald, Elizabeth G. Holliday, Jay C. Horvat, Anne E. Vertigan, Joe Van Buskirk, Geoffrey G. Morgan, Edward Jegasothy, Ivan Hanigan, Vanessa E. Murphy, Megan E. Jensen

**Affiliations:** 1 School of Medicine and Public Health, University of Newcastle, Newcastle, NSW Australia; 2 Asthma and Breathing Research Program, Hunter Medical Research Institute, Newcastle, NSW Australia; 3 Menzies Institute for Medical Research, University of Tasmania, Hobart, TAS Australia; 4 Tasmanian School of Medicine, University of Tasmania, Hobart, TAS Australia; 5 Department of Respiratory and Sleep Medicine, John Hunter Hospital, Newcastle, NSW Australia; 6 School of Nursing and Midwifery, University of Newcastle, Newcastle, NSW Australia; 7 School of Biomedical Sciences and Pharmacy, University of Newcastle, Newcastle, NSW Australia; 8 Department of Speech Pathology, John Hunter Hospital, Newcastle, NSW Australia; 9 Sydney School of Public Health, and University Centre for Rural Health, Faculty of Medicine and Health, University of Sydney, Sydney, NSW Australia

**Keywords:** Breast milk, Infant feeding, Environmental contaminants, Landscape fire, Bushfire, Smoke, Australia

## Abstract

**Background:**

The 2019/2020 Australian landscape fires (bushfires) resulted in prolonged extreme air pollution; little is known about the effects on breastfeeding women and their infants. This study aimed to examine the impact of prolonged landscape fires on infant feeding methods and assess the concentration of polycyclic aromatic hydrocarbons (PAHs) and elements in breast milk samples.

**Methods:**

From May – December 2020, women with asthma, who were feeding their infants during the fires, were recruited from an existing cohort. Data on infant feeding and maternal concern during the fires were retrospectively collected. Breast milk samples were collected from a sample of women during the fire period and compared with samples collected outside of the fire period for levels of 16 PAHs (gas chromatography coupled with mass spectrometry), and 20 elements (inductively coupled plasma-mass spectrometry).

**Results:**

One-hundred-and-two women who were feeding infants completed the survey, and 77 provided 92 breast milk samples. Two women reported concern about the impact of fire events on their infant feeding method, while four reported the events influenced their decision. PAHs were detected in 34% of samples collected during, versus no samples collected outside, the fire period (cross-sectional analysis); specifically, fluoranthene (median concentration 0.015 mg/kg) and pyrene (median concentration 0.008 mg/kg) were detected. Women whose samples contained fluoranthene and pyrene were exposed to higher levels of fire-related fine particulate matter and more fire days, versus women whose samples had no detectable fluoranthene and pyrene. Calcium, potassium, magnesium, sodium, sulphur, and copper were detected in all samples. No samples contained chromium, lead, nickel, barium, or aluminium. No statistically significant difference was observed in the concentration of elements between samples collected during the fire period versus outside the fire period.

**Conclusions:**

Few women had concerns about the impact of fire events on infant feeding. Detection of fluoranthene and pyrene in breast milk samples was more likely during the 2019/2020 Australian fire period; however, levels detected were much lower than levels expected to be related to adverse health outcomes.

**Supplementary Information:**

The online version contains supplementary material available at 10.1186/s13006-023-00550-8.

## Background

The 2019/2020 Australian landscape fire (bushfire) period was unprecedented in terms of severity, extent, and duration [1]. People living in Eastern Australia were affected by extreme air pollution for periods ranging from weeks to months [2, 3]. Landscape fire smoke is composed of a mixture of many pollutants with potential public health impacts, including fine particulate matter (PM_2.5_), carbon monoxide, polycyclic aromatic hydrocarbons (PAHs), reactive metals, and ozone [4–6]. Landscape fire can contaminate the environment in affected communities [7, 8] and has established adverse impacts on human health such as adverse pregnancy outcomes, increased paediatric respiratory care visits and increased hospital admission and emergency visits for respiratory and cardiovascular disease [3, 6, 9–11].

Vulnerable populations including pregnant women, breastfeeding women and their growing offspring, and children are at high risk of adverse outcomes following exposure to environmental contaminants [10, 12–16]. Studies have shown that exposure to environmental contaminants such as PAHs and metals increases the risk of adverse health outcomes in children including respiratory morbidity [17, 18], onset of asthma and asthma exacerbation [19]. However, the impact of the landscape fire events on breastfeeding women, and whether exposure to this extreme air pollution is associated with increased levels of pollutants in breast milk, remains unknown.

Breast milk contains macronutrients (fat, protein, and carbohydrate) and micronutrients required for infant health and development [20, 21]. Many of these elements have been reported to play essential roles in biological function and early development through their activity as antioxidants, enzyme cofactors and as components of hormones [22]. Conversely, environmental contaminants, such as PAHs and metals, can accumulate in breast milk [12, 15, 23–26]. If ingested in large amounts, PAHs and certain metals can potentially interfere with normal growth and development [18, 27, 28].

In addition to the global benefits of breastfeeding for mother and child, there is evidence indicating that breastfeeding has the potential to minimise the impacts of environmental contaminants through supporting the development of a strong immune system [29, 30]. However, research has demonstrated that breastfeeding/infant feeding regimes can be significantly impacted during natural disasters such as landscape fires and flood [31, 32]. A study of the effect of landscape fire evacuation on infant feeding in Canada indicated that exclusive breastfeeding rates were negatively impacted by landscape fire evacuation [32]. However, to our knowledge, no research has been published examining the impact of landscape fires on infant feeding methods in women with asthma in the Australian context. Therefore, in women with asthma, this study aimed to examine (a) the impact of the 2019/2020 Australian landscape fires on infant feeding methods; and (b) the concentration of PAHs and elements in breast milk samples collected during, versus outside (before or after), the landscape fire period.

M**ethods.**

### Study population

Participants with mild to moderate asthma from the Breathing for Life Trial (BLT) and Breathing for Life Trial-Nutrition study (BLT-NUT) were included in this study. BLT has been described previously [33]; briefly, pregnant women aged ≥ 18 years, with physician-diagnosed asthma and symptoms of asthma or use of asthma pharmacotherapy (β2-agonist, and/or inhaled corticosteroid in the past 12 months), and who were 12–23 weeks gestation at the time of randomization were enrolled across several sites in Eastern Australia.

### Survey

We conducted a retrospective, cross-sectional study to assess the impact of landscape fire smoke exposure in women with asthma during the 2019/2020 Australian fire period (1 October 2019 to 29 February 2020). The survey, results of which are reported elsewhere [34], collected information on general and asthma-specific symptoms, risk mitigation strategies and sources of information utilised during the fire period by mothers with asthma; the data relating to infant/toddler feeding is presented herein. A template of the tool was developed using REDCap (Research Electronic Data Capture) to capture the survey responses [35, 36]. The survey commenced on the 19^th^ of May 2020 and closed on the 2^nd^ of December 2020. Women were invited to complete the survey online, by phone, or on paper. Women who were feeding a child under 2 years during the fire period were prompted to complete an additional section on infant/toddler feeding. Women provided information on the feeding method(s) used during the landscape fire events, any concerns about infant/toddler feeding during the events, and whether the landscape fire events influenced their decisions around infant/toddler feeding, with options for free text. If they were feeding more than one child < 2 years of age, women were prompted to answer for the youngest child.

Participate residential address were used to estimate fire-related PM_2.5_ and fire days. Details are provided in the supplement (Additional file).

### Breast milk collection

In a longitudinal follow-up, a subset of BLTNUT participants (John Hunter Hospital site only, NSW), between January 2018 and July 2020, a volume of up to 25 mL of breast milk was collected, pre-feed, into a sterile container via manual/breast pump expressing, at one and/or two scheduled timepoints (early postpartum and 6 months postpartum). All samples were stored at -80^0^C prior to shipping to the Australian National Measurement Institute, Melbourne for analysis.

Demographic data, including maternal age, weight, height, smoking status and parity, and infant sex and age were obtained from the existing datasets. Maternal BMI (kg/m^2^) was calculated in pregnancy and classified as healthy weight [18.5 – 24.9], overweight [25 – 29.9], or obese [≥ 30].

### Sample preparation and procedure

#### Polycyclic Aromatic Hydrocarbons quantification

Tandem Gas Chromatography and Mass Spectrometry (GC–MS/MS) was used for the detection and quantification of the concentrations of 16 PAHs designated by US Environmental Protection Agency (EPA) in the list of priority pollutants (naphthalene, acenaphthylene, acenaphthene, fluorene, phenanthrene, anthracene, fluoranthene, pyrene, benzo[a]anthracene, chrysene, benzo[b]fluoranthene, benzo[k]fluoranthene, benzo[a]pyrene, indeno[1,2,3-cd]pyrene, dibenzo[a,h]anthracene and benzo[g,h,i]perylene) per mg of milk fat and the total PAH concentration was calculated [37]. Further detailed methods are given in the Additional file. The limit of detection (LOD) was 0.01 mg/kg breast milk.

### Element quantification

Using inductively coupled plasma-mass spectrometry (ICP-MS) or inductively coupled optical emission spectrometry (ICP-OES) [38], the concentration of 20 elements was quantified (aluminium, antimony, arsenic, barium, calcium, chromium, cobalt, copper, iron, lead, lithium, magnesium, manganese, molybdenum, nickel, potassium, selenium, sodium, sulphur, and vanadium). Further details on the methods are given in the Additional file. The LOD was 0.20–10.00 mg/kg milk for sodium, potassium, sulphur, iron, calcium, and magnesium, and ranged from 0.01–0.50 mg/kg milk for remaining elements.

#### Landscape smoke exposure data for women who provided breast milk samples

The landscape fire period was defined as 1 October 2019 to 29 February 2020 (the 2019/2020 black summer fire period in Eastern Australia). We obtained daily 24-h mean PM_2.5_ data from fixed-site government air quality monitoring stations within the Sydney greater metropolitan region (NSW Department of Planning, Industry and Environment) and identified landscape fire smoke days from a database based on government data and satellite imagery [39–42]. The measured daily data were interpolated within the study area using an inverse distance weighting procedure to estimate the daily PM_2.5_ (µg/m^3^) exposure concentration for each participant’s residential location [43].

Landscape fire days were defined as days when: (i) the regional 24-h average of PM_2.5_ concentration exceeded the 95^th^ percentile based on the period 01/01/2000 to 31/12/2018 for the study area; and (ii) there was visual confirmation of fire for that day, or up to 3 days before or after, via satellite imagery. Elevated PM_2.5_ levels on these days could then be attributed to landscape fire smoke [43]. To control for spatial variability in the region, an additional requirement was that the interpolated PM_2.5_ reading for each participant’s residential address also exceeded the 95^th^ percentile for the region.

For each participant, their daily PM_2.5_ concentration levels during the 152-days of the fire period were averaged to obtain their mean PM_2.5_ (µg/m^3^) exposure. The participant’s peak PM_2.5_ (µg/m^3^) exposure was determined as the maximum 24-h concentration value to which a participant was exposed during the 152-day fire period. Total fire days were calculated by summing all fire days over the period while maximum consecutive fire days obtained by counting the number of highest fire days in row over the period. Further information is provided in the Additional file.

Given that PM_2.5_ remains in the atmosphere for periods of hours to weeks (depending on their size) [44], and most PAHs have a short half-life ranging from hours to weeks [45], a seven day exposure period was selected for exposure outcomes.

### Statistical analysis

Data were graphically inspected for normality and checked using the Shapiro–Wilk normality test. Individual exposure values were assigned according to the women’s residential address. *For survey and samples*- data for continuous variables were summarized using mean with standard deviation (SD), or median with interquartile range (Q1, Q3). Data for categorical variables were summarized using frequency with percent. *For samples*- We estimated exposure as average and peak PM_2.5_ (µg/m^3^), total fire days and maximum consecutive fire days in the seven days prior to sample collection to assess the relationship between exposure to landscape fire smoke and the level of PAHs and elements in breast milk. *Group comparisons (samples)*—An independent t-test and Wilcoxon rank-sum test were performed to compare the distribution of elements between samples collected during, and outside, the fire period. The McNemar test was used to compare the proportion of samples with detectable PAHs and elements between samples collected during the fire period and outside the fire period. Paired t-tests and Wilcoxon signed-rank tests were used to test the within-person difference in concentrations of elements between samples collected during, versus outside, the fire period. *Correlations (samples)*—Spearman correlation coefficients were used to assess the correlation between fire-related PM_2.5_ or fire days and concentration of elements in samples with detectable concentrations. A *p*-value of < 0.05 was considered statistically significant. Statistical analysis was performed using R version 4.0.3 (Vienna, Austria) [46].

## Results

### Survey

The results related to respiratory and non-respiratory symptoms, asthma symptoms and exacerbation and mental health are reported elsewhere [34]; briefly, 82% participants experienced respiratory and non-respiratory symptoms and 85% asthma symptoms during the landscape fire period [34]. Furthermore, 86% reported having an asthma exacerbation during the fire period, with 20% reporting they started/increased OCS use for an asthma exacerbation(s) during the fire period. One-hundred-and-two women completed the infant feeding section of the survey. Women were a mean (SD) age of 33.6 (± 5.9) years. The mean age of their baby was 13.7 (± 6.4) months during the fire period. Over two-third (69%) had never smoked and 41% rated their general health as ‘Good’. Three (3%) women were evacuated during the fire period (Table 1).

**Table 1:** Characteristics of the women who completed the survey on infant feeding during the 2019/20 Australian landscape fire period (*n* = 102)

Variables	N (%)
Maternal age (years) Mean (SD)	33.6 (± 5.9)
Age of the baby during landscape fire period (months), Mean (SD)	13.7 (± 6.4)
Smoking status n (%)	
Never smoker	70 (68.6)
Ever smoker	26 (25.5)
Current smoker	6 (5.9)
General Health n (%)	
Excellent	11 (10.8)
Very good	37 (36.3)
Good	42 (41.2)
Fair	10 (9.8)
Poor	2 (2.0)
Diagnosed any new condition in the past 6 months n (%)	2 (2.0)
Had to leave residence/to evacuate during fire period n (%)	3 (2.9)

Women experienced a median daily average PM_2.5_ exposure over the fire period of 16.7 μg/m^3^ [16.4, 16.8] and median peak PM_2.5_ of 105.9 μg/m^3^ [99.6,111.8] (Additional File Table S1).

Of the 102 women who completed the infant feeding sections of the survey, 81% reported their infant/toddler received breast milk during the fire period, and 38% reported their infant/toddler was consuming solid foods. Only two (2%) women reported concern about the impact of landscape fire events on their infant feeding method, while three (3%) were unsure. Four (4%) women reported the fire events influenced their decision on how they fed their infant, with responses as follows: ‘stopped breastfeeding earlier than planned’ (*n* = 1), ‘increased breastfeeds’ (*n* = 1), ‘delayed the introduction of solids’ (*n* = 1), and ‘stopped breastfeeding outdoors’ (*n* = 1) (Table 2). Reported factors related to the landscape fire events that influenced their feeding decisions were ‘concerned about smoke exposure’ (*n* = 2), ‘breast milk supply affected’ (*n* = 2), ‘evacuation’ (*n* = 2) and ‘moved location’ (*n* = 1), ‘access to resources e.g. formula, bottles, sterilisers, nipple shields, feeding lines’ (*n* = 1), and ‘my infant/toddler experienced difficulty breastfeeding’ (*n* = 1). The free-text responses (presented in Additional File; Table S2) provided by women indicated that breastfeeding was difficult at times but also provided a positive influence during the fire period.

**Table 2:** Concerns about the effect of landscape fire events on infant feeding (*n* = 102)

Variables	Number (%)
How would you describe the way in which your infant/toddler was predominantly fed for the duration of the landscape fire period? More than one answer possible, *n* = 102	
Breast milk	83 (81.4)
Formula	13 (12.7)
Milk (animal sources e.g. cow’s or plant-based e.g. soy)	6 (5.9)
Solid foods	39 (38.2)
Other (breast milk and solid, express breast milk (EBM) and donor EBM)	2 (2.0)
Did you have any concerns about the impact of the landscape fire events on the way in which you chose to feed your baby (e.g. breastfeeding, pumping/expressing breast milk, formula / milk or milk alternative, solid foods)? *n* = 102	
Yes	2 (2.0)
No	97 (95.1)
Unsure	3 (2.9)
Were your **decisions** around how you fed your infant/toddler (e.g. breast milk, formula, milk or milk alternative, solid foods) influenced at all by the landscape fire? *n* = 102	
Yes	4 (3.9)
No	98 (96.1)

### Women who provided breast milk samples

**Fig. 1:**
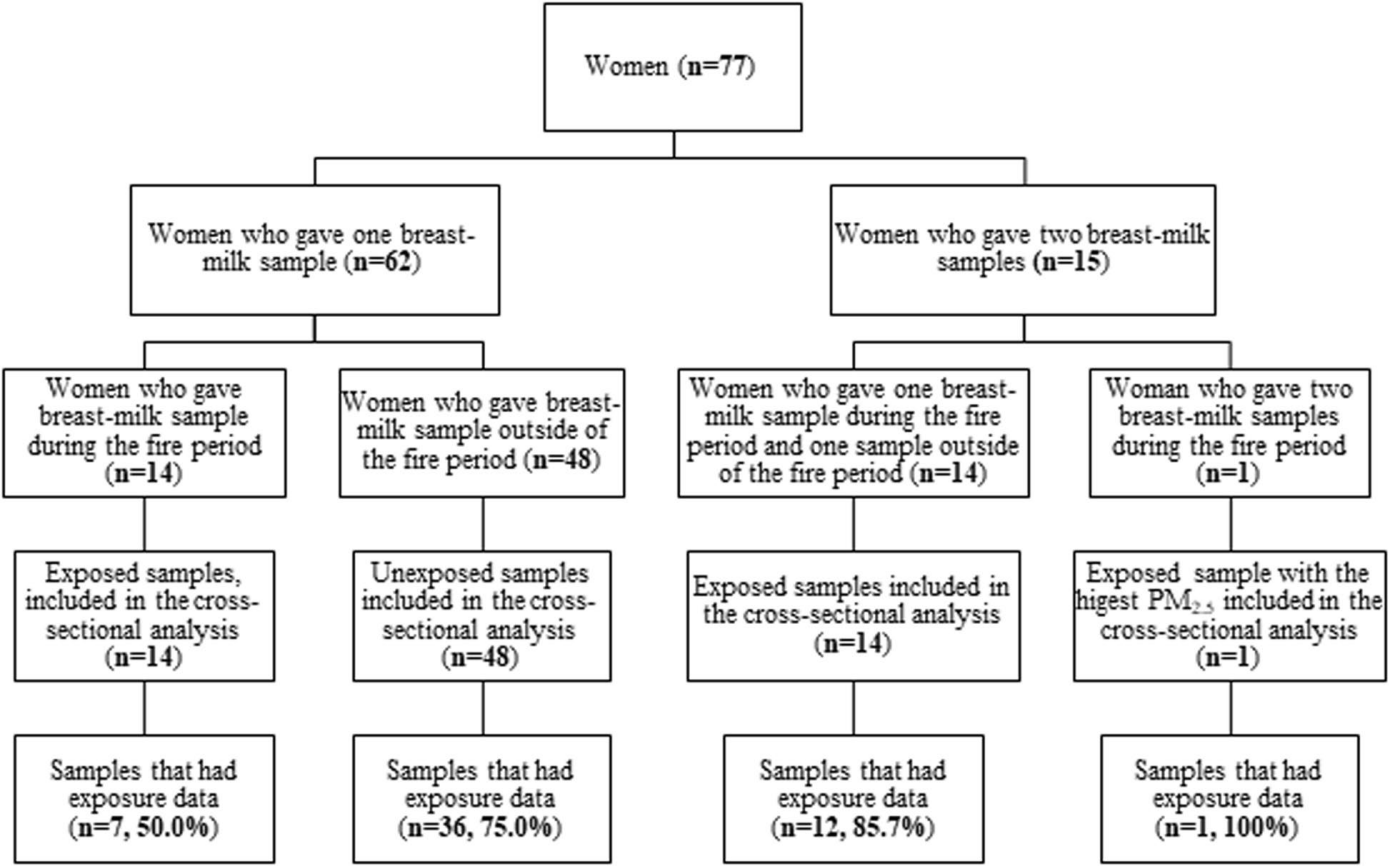
Participants flow diagram

Seventy-seven women provided a total of 92 breast milk samples during the study period (62 provided one sample; 15 provided two samples); of which 14 also completed the survey. The cross-sectional comparison included 77 samples (*n* = 29 during the fire period and *n* = 48 outside of the fire period), whilst the paired comparison included 28 samples from 14 women. (Fig. 1).

### General characteristics of women who provided breast milk samples

Participants had a median [Q1, Q3] age of 30 [28, 34] years and BMI of 27.6 [25.5, 33.5] kg/m^2^. Most (75%) participants were overweight or obese and 4% were current smokers. Fifty-seven (62%) samples were collected during the first postpartum appointment [median months at sample collection = 1.7 months (1.5, 1.9)], while 35 (38%) samples were collected during the second postpartum appointment [median months at sample collection = 6.3 months (6.1, 6.6)] (Table 3).

**Table 3:** General characteristics of women who provided breast milk samples (*n* = 77)

Participant’s characteristics	Values
Age (years)^a^	30 (28,34)
BMI (kg/m^2^) ^a^	27.6 (25.5, 33.5)
BMI category (%) ^b^	
Healthy weight	19 (24.7)
Overweight	30 (38.9)
Obese	28 (36.4)
Smoking status (%) ^b^	
Never smoker	52 (67.5)
Ex-smoker	22 (28.6)
Current smoker	3 (3.9)
Parity (%) ^b^	
1	40 (51.9)
≥ 2	37 (48.1)
Age of the baby at sampling (months) (92 samples	
Age at first infant follow-up appointment ^a^	1.7 (1.5, 1.9)
Age at second infant follow-up appointment ^a^	6.3 (6.1, 6.6)

^a^Median (Q1, Q3)

^b^count (%)

#### Exposure to PM_2.5_

**Fig. 2:**
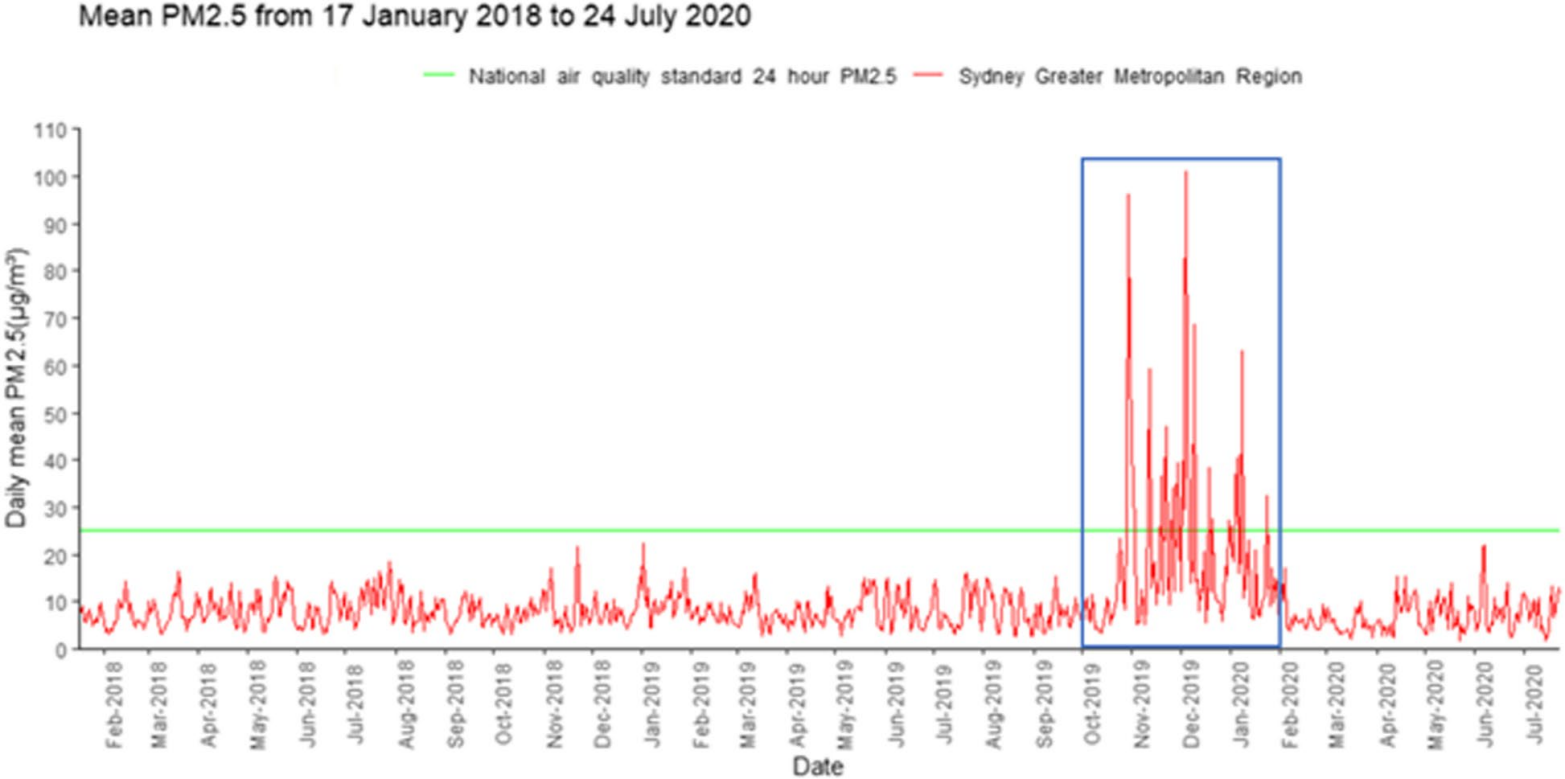
Population weighted mean PM_2.5_ concentration in Sydney Greater Metropolitan Region before, during and following the 2019/2020 fire period. The blue rectangle indicates the 2019/2020 Australian Black Summer fire period (1 October 2019 to 29 February 2020)

Most participants lived within Newcastle and the Hunter Region, which forms part of the Sydney greater metropolitan region for the purpose of air pollution measurements. Of the 77 women providing breast milk samples, 56 (73%) had exposure data. Twenty-one (27%) participants who lived outside of the Sydney greater metropolitan region had no exposure data. Figure 2 depicts population weighted daily average PM_2.5_ concentration in the study area before, during and following the 2019/2020 Australian fire period. On approximately 18% of days, the PM_2.5_ concentration during the fire period exceeded the national air quality 24-h standard of 25 μg/m^3^ (Fig. 2).

#### Landscape fire smoke exposure before sample collection

Of the 92-breast milk samples collected during the study period, only 56 (60.9%) had exposure data (20 (69.0%) breast milk samples collected during the fire period and 36 (75.0%) collected outside of the fire period had exposure data). The median daily average and peak PM_2.5_ exposure, and the median total fire days and maximum consecutive fire days, were statistically significantly higher during the 2019/2020 fire period than outside the fire period (Table 4).

**Table 4:** Landscape fire related PM_2.5_ exposure and fire days in the study period for women who provided breast milk samples in the Sydney Greater Metropolitan Region (*n* = 56)

Variables	Outside the fire period (*n* = 36)		During the fire period (*n* = 20)		*p*-value*
	Median (Q1, Q3)	Range	Median (Q1, Q3)	Range	
Average PM_2.5_(µg/m^3^)	7.7 (6.6, 10.2)	4.8 – 12.3	16.1 (9.6, 23.7)	5.0 – 40.6	< 0.001
Peak PM_2.5_ (µg/m^3^)	11.4 (10.0, 14.8)	7.9 – 16.6	31.9 (19.1, 59.4)	8.9 – 97.3	< 0.001
Fire days	0 (0,0)	0 – 2	0.5 (0,3.5)	0—6	< 0.001
Maximum consecutive fire days	0 (0,0)	0 – 1	0.5 (0,3)	0—11	< 0.001

^*^Wilcoxon rank-sum test

### Cross-sectional analysis for breast milk samples

#### Detected PAHs

**Fig. 3:**
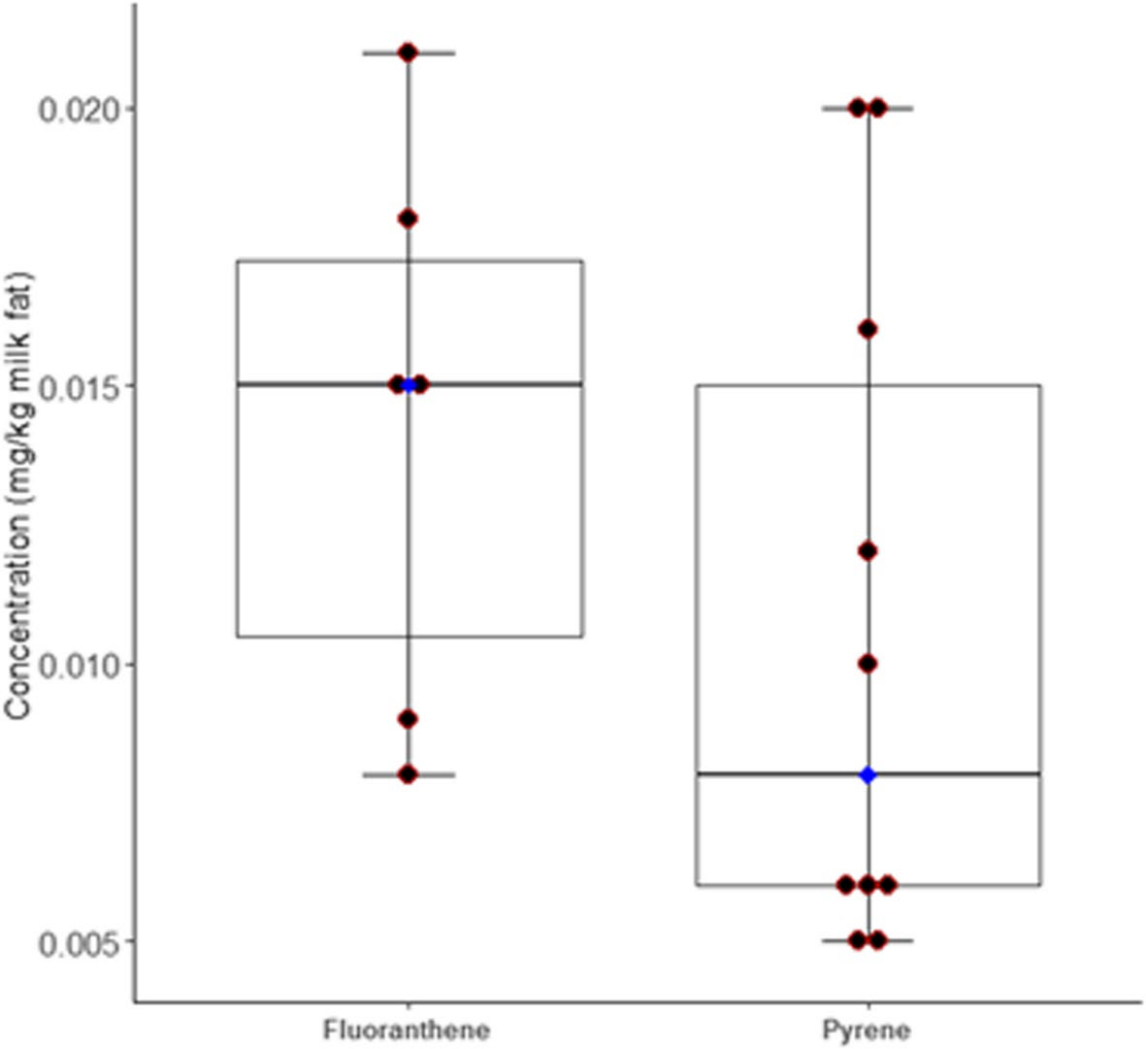
Concentrations of fluoranthene and pyrene detected in breast milk samples. The horizontal boundaries of the box indicate 25^th^ to 75^th^ percentile and the whiskers represent the highest and lowest values. The heavy horizontal line within the box or blue dot represents the median value

Two (13%) of the 16 screened PAHs were detected in the samples: fluoranthene and pyrene were detected in six (21%) and ten (34%) samples collected during the fire period, respectively. No PAHs were detected in samples collected outside the fire period. The median of fluoranthene and pyrene detected in breast milk samples were 0.015 mg/kg milk fat (range 0.008 to 0.021 mg/kg) and 0.008 mg/kg milk fat (range 0.005 to 0.020 mg/kg), respectively (Fig. 3). To understand the amount of fluoranthene and pyrene exposure to infants through breast milk, daily intake (mg/kg body weight/day) was calculated assuming 700 g/day average daily intake of milk by infant [12, 47], having 5 kg body weight [12, 48] and assumed 4.0% milk fat content [12, 49]. The following equation was used to estimate mean daily intake of fluoranthene and pyrene.

$$\textbf{Estimated daily intake}(\mathbf{m}\mathbf{g}/\mathbf{k}\mathbf{g}\mathbf{b}\mathbf{o}\mathbf{d}\mathbf{y}\mathbf{w}\mathbf{e}\mathbf{i}\mathbf{g}\mathbf{h}\mathbf{t}/\mathbf{d}\mathbf{a}\mathbf{y}) = [\text{Lipid content in milk }(\mathrm{\%})] [\mathrm{toxin }(\mathrm{PAHs})]\mathrm{ X }[\text{Milk consumption per day}]/\mathrm{kilogram body weight}.$$

Based on an infant of approximately 6 months of age, this may equate to a potential daily ingestion of up to 0.0001 mg/kg body weight per day for both fluoranthene and pyrene; this value is 400 times below levels cited in the literature as potentially causing harm (i.e. above 0.04 mg/kg body weight of fluoranthene and 0.03 mg/kg body weight of pyrene per day) [50].

#### Detected elements

Of the twenty elements analysed, 13 (65%) were detected in breast milk samples (Table 5). Calcium, potassium, magnesium, sodium, sulphur, and copper were detected in all samples. Selenium (79% and 92%), iron (79% and 85%), and manganese (3% and 8%) were detected in samples collected both during and outside the fire period, respectively. Three and two samples collected outside the fire period contained nickel and lead, respectively, whilst one sample contained chromium and barium. The median of elements concentration was in the range of World Health Organization (WHO) limit except for copper and manganese [51]. Formula for estimating daily intake of elements via breast milk is provided in additional file. The daily intake of copper and manganese was 0.01 and 0.31 mg/kg per day, respectively, this value is below levels expected to cause any adverse health effects in a child based on studies of these elements in drinking water ( i.e. 1 mg/kg body weight of manganese and 1.3 mg/kg body weight of copper in drinking water) [52, 53]. No statistically significant difference in elemental concentrations was observed between samples collected during versus outside the fire period (*p* > 0.05).

**Table 5:** Concentrations of elements detected in breast milk samples collected during, versus outside, the 2019/20 fire period (*n* = 77)

**Elements (mg/kg)**	**Outside the fire period (*n* = 48)**			**During the fire period (*n* = 29)**				**WHO Acceptable Ranges in Breast Milk (mg/kg)**	**Higher than WHO limit, n (%)**
	**Detected n (%)**	**Median (Q1, Q3)**	**Range**	**Detected n (%)**	**Median (Q1, Q3)**	**Range**	***p*-value**		
Calcium	48 (100)	270 (250,305)	210 – 380	29 (100)	280 (260,310)	190—350	0.81#	220—300	21 (27.3)
Potassium	48 (100)	500 (450,530)	370 – 780	29 (100)	490 (460,530)	400—630	0.98*	410—550	10 (13.0)
Magnesium	48 (100)	32 (27.5,36.0)	16—46	29 (100)	30 (26,34)	18 – 43	0.21#	29—38	10 (13.0)
Sodium	48 (100)	100 (79.5,130)	33 – 220	29 (100)	93 (69,130)	41—400	0.49*	90—130	17 (22.1)
Sulphur	48 (100)	120 (100,130)	87 – 200	29 (100)	120 (100,130)	90 -160	0.45*	-	-
Copper	48(100)	0.35 (0.24,0.61)	0.07 – 12.0	29 (100)	0.56 (0.35, 0.74)	0.04—1.3	0.07*	0.18—0.31	52 (67.5)
Iron	41 (85.4)	0.24 (0.18,0.31)	0.12 – 0.57	23 (79.3)	0.30 (0.21, 0.39)	0.11 – 0.63	0.13*	0.35 – 0.72	0
Selenium	44 (91.7)	0.01 (0.01, 0.02)	0.01 – 0.03	23 (79.3)	0.01 (0.01, 0.02)	0.01 – 0.03	0.64*	0.01 – 0.02	4 (6.0)
Manganese	4 (8.3)	0.01 (0.01 – 0.02)	0.01 – 0.02	1 (3.4)	-	0.01 – 0.01	NA	0.003—0.004	5 (100)

^*^Wilcoxon rank-sum test, # t-test, NA: Not applicable, because there was no positive sample detected during the fire period or does not allow for statistical analysis

### Paired analysis

Of the 14 paired samples, fluoranthene was detected in one sample collected during the fire period and one sample collected outside the fire period (concentration 0.02 mg/kg versus 0.01 mg/kg, respectively), while pyrene was detected in four (29%) samples collected during the fire period only (median 0.01 mg/kg, range 0.01 – 0.02); no statistically significant within-person differences were observed (*p* > 0.05) (Additional File; Table S3).

Calcium, potassium, magnesium, sodium, sulphur, and copper were detected in all paired samples (Table 6). Manganese and aluminium were each detected in one sample only, collected outside of the fire period (concentrations 0.01 and 0.68 mg/kg, respectively). No statistically significant within-person difference was observed in the concentration of elements between samples collected during, versus outside, the fire period (*p* > 0.05).

**Table 6:** Concentrations of elements detected in paired breast milk samples collected during the 2019/20 fire period and outside the fire period (*n* = 14 paired samples)

**Elements (mg/kg)**	**Outside the fire period (14 samples)**			**During the fire period (14 samples)**				**WHO Acceptable Ranges in Breast Milk (mg/kg)**	**Higher than the WHO ranges, n (%)**
	**Detected, n (%)**	**Median (Q1, Q3)**	**Range**	**Detected, n (%)**	**Median (Q1, Q3)**	**Range**	***p*- value**		
Calcium	14 (100)	295 (250,340)	220 – 350	14 (100)	265 (240,310)	190 – 350	0.15^ ≠^	220—300	10 (35.7)
Potassium	14 (100)	510 (450,600)	390 – 660	14 (100)	480 (440,540)	400 – 560	0.08^†^	410—550	6 (21.4)
Magnesium	14 (100)	29 (26,34)	23 – 40	14 (100)	32 (25,36)	19 – 40	0.62 ^≠^	29—38	3 (10.7)
Sodium	14 (100)	93 (64,110)	51 – 420	14 (100)	77 (65,130)	41 -170	0.53^†^	90—130	5 (17.8)
Sulphur	14 (100)	125 (110,130)	77 – 180	14 (100)	105 (99,120)	90 – 130	0.12^†^	-	-
Copper	14 (100)	0.67(0.36,0.9)	0.2 – 1.0	14 (100)	0.55 (0.41,0.77)	0.04 – 1.0	0.46^†^	0.18—0.31	22 (78.6)
Iron	12 (85.7)	0.25 (0.22,0.29)	0.12 – 0.52	11 (78.6)	0.31 (0.21,0.35)	0.18 – 0.48	0.23^†^	0.35 – 0.72	0
Selenium	10 (71.4)	0.02 (0.01,0.02)	0.01 – 0.02	11 (78.6)	0.01 (0.01,0.02)	0.01 – 0.02	0.05^†^	0.01 – 0.02	1 (3.6)

^≠^ paired t-test ^†^ Wilcoxon signed-rank test

### Landscape fire smoke exposure, and PAHs in breast milk samples

The median daily average PM_2.5_, peak PM_2.5,_ total fire days and maximum consecutive fire days were significantly higher for women whose breast milk samples contained detectable fluoranthene and pyrene (both *p* < 0.005), compared to women whose breast milk samples had no detectable fluoranthene or pyrene (Table 7).

**Table 7:** Bivariate analysis of landscape fire smoke exposure, and PAHs in breast milk samples (*n* = 56)

Exposure data available	**Fluoranthene in breast milk sample**			**Pyrene in breast milk sample**		
(*n* = 56)	**Detected (*n* = 2)**	**Not detected (*n* = 54)**	***p*-values** *	**Detected (*n* = 4)**	**Not detected (*n* = 52)**	***p*-values** *
Average PM_2.5,_ µg/m^3a^	30.3 (20.0, 40.6)	8.6 (6.8, 12.0)	0.026	23.3 (16.0, 33.6)	8.2 (6.7,11.9)	0.004
Peak PM_2.5_, µg/m^3a^	77.1 (56.8, 97.3)	14.1 (10.3, 18.4)	0.012	59.9 (39.8, 80.2)	13.8 (10.2, 17.1)	0.001
Total fire days^a^, n	3 (2,4)	0 (0,0)	0.045	2 (1,3)	0 (0,0)	0.035
Maximum consecutive fire days^a^, n	3 (2,4)	0 (0,0)	0.043	2 (1,3)	0 (0,0)	0.031

^a^median (Q1, Q3), *Wilcoxon rank-sum exact test

There was no statistically significant difference in median values for average PM_2.5_, peak PM_2.5,_ total fire days and maximum consecutive fire days, between breast milk samples with versus without detectable levels of manganese, lead, and nickel (*p* > 0.05) (Additional File; Table S4).

### Correlation between landscape fire smoke related exposure measures and element concentrations in breast milk samples

For the elements calcium, potassium, magnesium, sodium, sulphur, copper, iron, and selenium, no significant correlations were observed between landscape fire smoke exposure metrics and element concentration (*p* > 0.05) (Additional File; Table S5).

## Discussion

This is the first study to examine the impact of a prolonged landscape fire event on infant feeding methods amongst women with asthma and is the first to quantify and compare the concentration of PAHs and elements in breast milk samples collected during, versus outside, the 2019/2020 Australian fire period. Our study indicated that very few women had concerns about the impact of landscape fire events on infant/toddler feeding. Based on the cross-sectional analysis, PAHs were detected in 34% of samples collected during, versus no samples collected outside, the fire period. In a paired analysis, PAHs were detected in 29% of samples collected during the fire period and one (7%) sample collected outside the fire period. However, the concentrations of PAHs (fluoranthene and pyrene) detected were at very low concentrations and well below levels of concern to human health. These findings are reassuring, indicating that exposure during the fire period was not of immediate concern to infant feeding safety or a major influence on feeding methods in this sample population of women with asthma.

In our sample population, very few women reported concern about the impact of landscape fire events on their infant/toddler feeding method (2% concerned, 3% were unsure); however, 4% reported the fire events influenced their decision on how they fed their infant/toddler. A study of the impact of a large-scale landscape fire evacuation on infant feeding in 115 women in Canada found that exclusive breastfeeding rates reduced from 64 to 36% following the fire evacuation and the use of infant formula increased [32]. Furthermore, women who continued breastfeeding during evacuation reported that breastfeeding was a source of comfort for their infants and gave them a sense of safety [32]. Likewise, free-text responses from our sample population, where evacuation occurred for 3% of women, also indicated breastfeeding provided comfort for mother and child during the fires. Based on our data and other studies women should be supported to continue breastfeeding during the fire period. Future research is needed to understand the impact of landscape fire events on infant feeding practices in women with asthma, particularly in more intensely affected areas including those that are evacuated during extreme and prolonged landscape fires.

Our study showed that the proportion of breast milk samples positive for fluoranthene and pyrene were higher during the fire period compared to outside the fire period. This is in line with previous reports of higher PAH concentrations in breast milk samples from women who reside in a highly industrialized locality [54], with reported levels reaching 0.4 mg/kg of breast milk; albeit these are concentrations drastically higher than those detected in our study. Furthermore, our findings suggest that higher landscape fire-related PM_2.5_ or fire days were linked to fluoranthene and pyrene in breast milk samples. Indeed, PAHs, including fluoranthene and pyrene, as well as naphthalene, acenaphthene, fluorene, phenanthrene, and anthracene have been associated with landscape fire pollution [55]. Reassuringly, we did not detect these additional PAHs in the samples (naphthalene, acenaphthene, fluorene, phenanthrene, anthracene). A report from the Hazelwood coal mine fire in Australia showed that the concentration of fluoranthene (1.5 ng/m^3^) and pyrene (2.4 ng/m^3^) in the atmosphere were higher during a coal mine fire period compared to the post mine fire period where they were not detectable [56]. It is likely that most of the PAHs are bound to PM_2.5_ [57, 58] suggesting that exposure predominantly occurs by inhalation of fire-derived PM.

Our findings showed that the maximum concentration of fluoranthene and pyrene in breast milk was 0.02 mg/kg. This might equate to a potential maximum daily ingestion of up to 0.0001 mg/kg body weight for both fluoranthene and pyrene; this is well below levels of concern to human health. The US EPA has set a regulation to protect human health from adverse effects of PAHs and suggested that ingestion of 0.04 mg/kg body weight of fluoranthene and 0.03 mg/kg body weight of pyrene per day is not likely to cause any harmful health effects [50]. The concentration of fluoranthene and pyrene detected in our study are extremely lower than the levels likely to be associated with adverse effect on health, which for fluoranthene and pyrene are 100 mg/kg/day and 75 mg/kg/day, respectively, in animal studies [59, 60]. The concentration of fluoranthene and pyrene detected in our study is also at the lower end of concentrations reported in previous exposure studies, where the maximum concentration of fluoranthene and pyrene in breast milk ranged from 0.01 to 0.12 mg/kg and 0.01 to 0.09 mg/kg, respectively [12, 54, 61]. Based on this information, the detection of extremely low concentrations of fluoranthene and pyrene in breast milk during extreme fire events in our sample population presents negligible risk to infant feeding safety and provides reassuring data previously unavailable. More research is warranted investigating more severely affected areas, or areas where there is high outdoor air pollution (e.g. heavy traffic road), which may give different results.

Elements that have been associated with adverse health outcomes, that is lead, nickel, aluminium, barium, and chromium, were not detected in samples collected during the fire period. As expected, calcium, potassium, magnesium, sodium, sulphur, and copper were detected in all breast milk samples, but there was no difference in any elements based on exposure. More than half of the samples had copper levels above the acceptable range recommended by the WHO; however the levels are similar to other results reported from Sweden, Greece, Spain and Portugal [62–65], ranging from 0.37 mg/kg to 0.50 mg/kg. In addition, manganese, although detected in less than 10% of samples, was detected at a higher level than the recommended acceptable range in breast milk by the WHO. The median manganese concentration in the present study was also higher (0.01 mg/kg) than that reported in other studies (0.003 to 0.006 mg/kg) [62, 66]. However, the daily intake of manganese and copper in the present study were lower than the level expected to cause any adverse health outcomes [52, 53]. A study of Australian landscape fires between 1994 and 2004, found that the concentration of trace elements such as lead and cadmium in the air were doubled during landscape fire periods compared to periods before or after, but not at levels of concern to human health [5]. Other studies have also reported that air pollution, including from fires, increases metals in the environment [56, 67]. A cross-sectional study in Spain [68] reported that women who live in high motor vehicle traffic areas and consume potatoes had lead concentrations (15.6 µg/L) in their breast milk exceeding the limits recommended by the WHO (2 –5 µg/L) [51, 69]. Authors also reported that women who smoke during pregnancy had cadmium in breast milk [68]. This study assessed long term chronic exposure to environmental contaminants while our study assessed a high exposure acute event, which may account for the differences in findings. Our results suggest that despite likely increases in contaminants in the atmosphere due to landscape fire smoke, this did not translate into high levels of contaminants in breast milk in our sample population.

Our study has limitations. There was a delay between 3 to 6 months between the end of fire period and the time of the survey, which may result in a recall bias. The small sample size in our study may have limited statistical power for assessing the relationship between landscape fire smoke related exposure data, and the concentration of PAHs and elements in breast milk with other confounders, such as smoking. Another limitation was the lack of information on participants’ lifestyle and environmental factors known to influence level of exposure to contaminants, including diet, water quality, indoor air pollution (e.g. wood burn), and time spent outdoors during the fire period, which may have influenced the level of PAHs and elements in their breast milk. In addition, our study participants were women with asthma so the findings of this study might be influenced by participants' behavioural changes such as staying at home and cleaning their living area more often to avoid allergens due to fear of contracting COVID-19 [70]. Research into proximity of women’s residential address to heavily fire affected areas, and those evacuated, might give different results than our study. Therefore, future studies with larger sample sizes, including information on lifestyle factors, are needed to further understand the impact of environmental exposures such as landscape fires on infant feeding practices and the concentration of PAHs and elements in breast milk.

## Conclusion

Despite prolonged exposure to landscape fire smoke, few women had concerns or changed the way they fed their infant during the 2019/20 Australian landscape fires. While detection of fluoranthene and pyrene in breast milk samples was more likely during these events, compared to outside the fire period, these contaminants were detected at concentrations unrelated to human health concerns. Women should be supported to continue breastfeeding during extreme air pollution events to protect maternal and child health. Further research is needed in more severely fire affected area to assess the impact of landscape fire on maternal concerns and infant feeding practices, and to examine the presence of contaminants in breast milk.

## Supplementary Information

**Additional file 1.**

## Data Availability

All data generated during this study are included in the manuscript and supporting files.
