# Supplementary material for: The impact of the 2019/2020 Australian landscape fires on infant feeding and contaminants in breast milk in women with asthma
Source: Int Breastfeed J. 2023 Feb 23;18:13. doi: 10.1186/s13006-023-00550-8 (PMC9947434; doi:10.1186/s13006-023-00550-8)
Supplement: Supplementary file 1 — Additional file 1. [file 13006_2023_550_MOESM1_ESM.docx]

**Additional file**

**Methods**

**Landscape fire smoke exposure for samples and women who completed the survey**

The exposure period was defined as 1 October 2019 to 29 February 2020 (the 2019/2020 black summer landscape fire period in Australia). We obtained daily 24-hour mean PM_2.5_ data from fixed-site government air quality monitoring stations within the Sydney greater metropolitan region (NSW Department of Planning, Industry and Environment) and identified landscape fire smoke days from a database based on government data and satellite imagery [1-4]. The measured daily data were interpolated within the study area using an inverse distance weighting procedure to estimate the daily PM_2.5_ (µg/m³) exposure concentration for each participant’s residential location [5].

Landscape fire days were defined as days when: (a) the regional 24-hour average of PM_2.5_ concentration exceeded the 95^th^ percentile based on the period 01/01/2000 to 31/12/2018 for the study area; and (b) there was visual confirmation of fire for that day, or up to 3 days before or after, via satellite imagery. Elevated PM_2.5_ levels on these days could be attributed to landscape fire smoke [5]. To control for spatial variability in the region, an additional requirement was that the interpolated PM_2.5_ reading for each participant’s residential address also exceeded the 95^th^ percentile for the region.

*For survey*- For each participant, their daily PM_2.5_ concentration levels over the 152-days of the landscape fire period were averaged to obtain their mean PM_2.5_ (µg/m³) exposure. The participant’s peak PM_2.5_ (µg/m³) exposure was determined as the maximum 24-hour concentration value to which a participant was exposed during the 152-day landscape fire period.

*For samples* - Given that PM_2.5_ remains in the atmosphere for periods of hours to weeks (depending on their size) [6], and most PAHs have a short half-life ranging from hours to weeks [7], a seven-day exposure period was selected for exposure outcomes.

**Sample preparation and procedure**

**Polycyclic Aromatic Hydrocarbons quantification**

Approximately 1 g of homogenised sample was mixed with water and acetonitrile, then extracted with the Quick, Easy, Cheap, Effective, Rugged, and Safe (QuEChERS) extraction kit (**Agilent Technologies,** USA). The extracts were cleaned by QuEChERS dispersive Solid Phase Extraction (dSPE) tubes, and the organic layer transferred into a GC vial and analysed by GC-MS/MS. Individual PAHs were separated by GC using a HP-5 MS column and detected with a GC mass spectrometer. The identity of separate PAHs was confirmed by comparing retention times and the ion ratios of each of the PAH standards against the peaks of the test sample extract at a given retention window and quantified by multi-point calibration of target analyte standards. Matrix matched calibration standards were employed to minimize matrix interferences. Each batch included a control blank, blank matrix, and a sample analysed in duplicate and sample recovery for every 10^th^ or last sample (if the batch was less than 10). The recovery was carried out by spiking a known concentration of PAH mix in a blank matrix.

**Elements quantification**

The sample was homogenized and a sub-sample (0.20 - 0.50g) was digested with re-distilled nitric acid (hydrochloric acid was included for the digestion of iron and aluminium) on a DigiPrep block for one hour. Samples were then transferred to a Milestone microwave to be further digested. After making up to the appropriate volume with Milli-Q (high purity) water, the digest was analysed for 20 elements (aluminium, antimony, arsenic, barium, calcium, chromium, cobalt, copper, iron, lead, lithium, magnesium, manganese, molybdenum, nickel, potassium, selenium, sodium, sulphur, and vanadium).

**Estimating daily intake of PAHs or elements via breast milk**

To understand the amount of PAHs or elements exposure to infants through breast milk, daily intake (mg/kg body weight/day) [8-10] was calculated assuming 700 g/day (0.7 kg/day) average daily intake of milk by infant [11], having 5 kg body weight [12, 13] and assumed 4.0% milk fat content [13, 14]. The daily intakes were calculated using the following formula:

**Estimated daily intake (mg/kg body weight/day)** = [Lipid content in milk (%)] [PAHs or elements)] X [Milk consumption per day]/kilogram body weight.

**Results**

**Table S1** Landscape fire smoke exposure data for women who completed the survey (**n=74**)

| **Variables** | Median [Q1, Q3] |
| --- | --- |
| Average PM_2.5_ (ug/m^3^) | 16.7 (16.4, 16.8) |
| Peak PM_2.5_ (ug/m^3^) | 105.9 (99.6,111.8) |
| Total fire days | 42 (41,43) |
| Maximum consecutive fire days | 11 (10,11) |

**Table S2** Free text response of women who were feeding their infants during landscape fire events (**n=102**)

| Survey questions | Participants’ response (free text responses) |
| --- | --- |
| Did you have any **concerns** about the impact of the bushfire events on the way in which you chose to feed your baby?  Please provide further information related to feeding your infant/toddler during the bushfire events, if you would like | Transfer of PM_2.5_ particles in breast milk. Unsure if bushfire pollution impacted quality of milk being passed on (32-year-old woman) |
|  | Hard to breastfeed on smoky days, baby not interested in attachment (37-year-old woman) |
|  | Feeding during events kept her more calm (39-year old woman) |
|  | During the bushfires I was only feeding my toddler once a day, in the evenings. I found the time with her calming and connecting. Sometimes the pressure of her body against mine put extra pressure on my breathing but nothing I wasn't used to and couldn't handle (33-year-old woman). |
|  | I used probiotics and vitamins during the bushfire season (30-year woman) |
|  | I had very little smoke around my house during the whole season. Except for one night the fire came to my house. It was thick black smoke, but I was evacuated within 10 minutes. The stress affected me, but I didn't notice my asthma being affected. My milk supply might have been down temporarily, due to stress I imagined. (42-year woman) |
|  | I was happy to be breastfeeding during the bushfire season (33-year-old woman) |
|  | I had to sit in the car with 2 and air con on to breastfeed to stay out of smoky air sometimes (24-year-old woman). |
|  | Continued breastfeeding toddler as normal (32-year-old woman) |
|  | Worried about bringing baby home from hospital with all the smoke at the time (25-year-old woman) |
|  | I moved very quickly to formula feeding as I had a lack of supply, so the bushfires were not a consideration in my case (36-year-old woman) |
|  | Up to five milk feeds in 24 hours, but feeds dropped rapidly in favour of solids during the bushfire period (baby-led). (40-year-old woman) |
|  | We were in the process of weaning from breast milk to formula as I was returning to work  (29-year-old woman) |

**Women who provided breast milk samples**

**Paired analysis**

**Table S3** Concentrations of PAHs detected in paired breast milk samples collected during the 2019/20 fire period and outside the fire period (**n=14 paired samples**).

| **PAHs (mg/kg)** | **Outside the fire period**  **(14 samples)** | | **During the fire period**  **(14 samples)** | |  |
| --- | --- | --- | --- | --- | --- |
|  | **Detected, n (%)** | **Range** | **Detected, n (%)** | **Range** | **p-value⸸** |
| Fluoranthene | 1 (7.1) | - | 1 (7.1) | - | 1.0 |
| Pyrene | 0 (0.0) | - | 4 (28.6) | 0.01 – 0.02 | 0.12 |

**⸸** McNemar exact test

**Table S4** Bivariate analysis of landscape fire smoke exposure, and manganese, lead, and nickel in breast milk samples collected from women with asthma during the fire period (**n=56**)

| Exposure | **Manganese** | | | **Lead** | | | **Nickel** | | |
| --- | --- | --- | --- | --- | --- | --- | --- | --- | --- |
| N=56 | **Detected (n=4)** | **Not detected (n=52)** | **p-value*** | **Detected (n=2)** | **Not detected (n=54)** | **p-value*** | **Detected (n=3)** | **Not detected (n=53)** | **p-value*** |
| Average PM_2.5,_ µg/m³^a^ | 6.5  (5.6, 8.7) | 9.3  (6.9, 12.3) | 0.09 | 6.1  (5.2, 7.0) | 9.3  (6.9, 12.2) | 0.10 | 7.0  (5.1, 10.6) | 9.3  (6.9, 12.2) | 0.30 |
| Peak PM_2.5,_ µg/m³^a^ | 10.8  (9.5, 13.3) | 14.5  (10.6, 22.0) | 0.22 | 10.4  (9.1, 11.7) | 14.5  (10.4, 21.2) | 0.29 | 11.7  (9.1, 14.1) | 14.7  (10.4, 21.2) | 0.36 |
| Fire days^a^, n | 0 (0,0) | 0 (0,0) | 0.74 | 0 (0,0) | 0 (0,0) | 1.0 | 0 (0,0) | 0 (0,0) | 0.95 |
| Maximum consecutive fire days^a^, n | 0 (0,0) | 0 (0,0) | 0.74 | 0 (0,0) | 0 (0,0) | 1.0 | 0 (0,0) | 0 (0,0) | 0.95 |

^a^ median (Q1, Q3), *Wilcoxon rank-sum exact test

**Table S5** Spearman correlations between exposure to PM_2.5_ or fire days and elements (n=77).

| **Elements** (mg/kg) | **Average PM_2.5_** | | **Peak PM_2.5_** | | **Bushfire days** | | **Maximum consecutive fire days** | |
| --- | --- | --- | --- | --- | --- | --- | --- | --- |
|  | **Rho** | **p-value** | **Rho** | **p-value** | **Rho** | **p-value** | **rho** | **p-value** |
| Calcium | -0.02 | 0.86 | -0.05 | 0.71 | -0.10 | 0.47 | -0.09 | 0.48 |
| Potassium | -0.04 | 0.79 | -0.09 | 0.51 | 0.01 | 0.93 | 0.02 | 0.90 |
| Magnesium | 0.17 | 0.20 | 0.10 | 0.45 | 0.11 | 0.41 | 0.10 | 0.44 |
| Sodium | -0.04 | 0.74 | -0.16 | 0.24 | -0.01 | 0.94 | -0.01 | 0.89 |
| Sulphur | -0.11 | 0.40 | -0.17 | 0.20 | 0.03 | 0.83 | 0.030 | 0.82 |
| Copper | 0.01 | 0.93 | 0.13 | 0.33 | 0.08 | 0.54 | 0.09 | 0.51 |
| Iron | 0.25 | 0.05 | 0.2 | 0.09 | 0.17 | 0.22 | 0.17 | 0.21 |
| Selenium | -0.02 | 0.87 | -0.06 | 0.66 | 0.002 | 0.98 | -0.002 | 0.99 |

References

1. Hourly site average pollutant concentration and meteorological data for 2018-2019. [<https://www.dpie.nsw.gov.au/air-quality/air-quality-concentration-data-updated-hourly>]

2. Hourly site average pollutant concentration data for 1994-2018. [ <https://www.dpie.nsw.gov.au/air-quality/search-for-and-download-air-quality-data>.]

3. Riley M, Kirkwood J, Jiang N, Ross G, Scorgie Y. Air quality monitoring in NSW: From long term trend monitoring to integrated urban services. Air Quality and Climate Change. 2020; 54(1):44–51.

4. Van Buskirk J, Hanigan I. 2021 Bushfire specific PM25 surface at participant’s residential locations for 2006-2020.Downloaded from the Centre for Air pollution, energy and health Research <https://cloudstor.aarnet.edu.au/plus/f/5638670382>. .

5. Johnston FH, Hanigan IC, Henderson SB, Morgan GG, Portner T, Williamson GJ *et al*. Creating an integrated historical record of extreme particulate air pollution events in Australian cities from 1994 to 2007. J Air Waste Manag Assoc. 2011; 61(4):390-398.

6. Health risks of particulate matter from long-range transboundary air pollution. Copenhagen : WHO Regional Office for Europe [ <https://apps.who.int/iris/handle/10665/107691>]

7. Motorykin O, Santiago-Delgado L, Rohlman D, Schrlau JE, Harper B, Harris S *et al*. Metabolism and excretion rates of parent and hydroxy-PAHs in urine collected after consumption of traditionally smoked salmon for Native American volunteers. Sci Total Environ. 2015; 514:170-177.

8. United States Environmental Protection Agency. Drinking Water Health Advisory for Manganese, EPA-822-R-04-003, U.S. EPA, Washington, DC, 2004. Available : <https://www.epa.gov/sites/production/files/2014-09/documen> ts/support_cc1_magnese_dwreport_0.pdf. Access date : 13 January 2023. .

9. Motas M, Jiménez S, Oliva J, Cámara M, Pérez-Cárceles MD. Heavy Metals and Trace Elements in Human Breast Milk from Industrial/Mining and Agricultural Zones of Southeastern Spain. Int J Environ Res Public Health. 2021; 18:9289.

10. Mitchell EJ, Frisbie SH, Roudeau S, Carmona A, Ortega R. Estimating daily intakes of manganese due to breast milk, infant formulas, or young child nutritional beverages in the United States and France: Comparison to sufficiency and toxicity thresholds. J Trace Elem Med Biol. 2020; 62:126607.

11. Institute of Medicine (US) Committee on Nutritional Status During Pregnancy and Lactation. Nutrition During Lactation. Washington (DC): National Academies Press (US). 1991.

12. Van Oostdam J, Gilman A, Dewailly E, Usher P, Wheatley B, Kuhnlein H *et al*. Human health implications of environmental contaminants in Arctic Canada: a review. Sci Total Environ. 1999; 230(1-3):1-82.

13. Acharya N, Gautam B, Subbiah S, Rogge MM, Anderson TA, Gao W. Polycyclic aromatic hydrocarbons in breast milk of obese vs normal women: Infant exposure and risk assessment. Sci Total Environ. 2019; 668:658-667.

14. Arcus-Arth A, Krowech G, Zeise L. Breast milk and lipid intake distributions for assessing cumulative exposure and risk. Journal of Exposure Science & Environmental Epidemiology. 2005; 15(4):357-365.
